# Cluster Analyses Reveals Subgroups of Children With Suspected Auditory Processing Disorders

**DOI:** 10.3389/fpsyg.2019.02481

**Published:** 2019-11-15

**Authors:** Mridula Sharma, Suzanne C. Purdy, Peter Humburg

**Affiliations:** ^1^Department of Linguistics, Australian Hearing Hub, Macquarie University, Macquarie Park, NSW, Australia; ^2^The HEARing CRC, Audiology, Hearing and Speech Sciences, The University of Melbourne, Parkville, VIC, Australia; ^3^Speech Science, School of Psychology, The University of Auckland, Auckland, New Zealand; ^4^Eisdell Moore Centre for Hearing and Balance Research, The University of Auckland, Auckland, New Zealand; ^5^Faculty of Human Sciences, Macquarie University, Macquarie Park, NSW, Australia

**Keywords:** auditory processing disorders, cluster, subgroup, memory, attention, reading, language

## Abstract

**Background:**

Some children appear to not hear well in class despite normal hearing sensitivity. These children may be referred for auditory processing disorder (APD) assessment but can also have attention, language, and/or reading disorders. Despite presenting with similar concerns regarding hearing difficulties in difficult listening conditions, the overall profile of deficits can vary in children with suspected or confirmed APD. The current study used cluster analysis to determine whether subprofiles of difficulties could be identified within a cohort of children presenting for auditory processing assessment.

**Methods:**

Ninety school-aged children (7–13 years old) with suspected APDs were included in a cluster analysis. All children had their reading, language, cognition and auditory processing assessed. Parents also completed the Children’s Auditory Performance Scale (CHAPS). Cluster analysis was based on tasks where age-norms were available, including word reading (Castles and Coltheart irregular and non-words test), phonological awareness (Queensland University Inventory of Literacy), language [Comprehensive Language of Assessment-4, Comprehensive Assessment of Spoken Language (CASL)], sustained attention (Continuous Performance Test), working memory (digits forward and backward), and auditory processing [Frequency Pattern Test (FPT), Dichotic Digits Test (DDT)]. Hierarchical cluster analysis was undertaken to determine the optimal number of clusters for the data, followed by a k-means cluster analysis.

**Results:**

Hierarchical cluster analysis suggested a four-group solution. The four subgroups can be summarized as follows: children with (1) global deficits, *n* = 35; (2) poor auditory processing with good word reading and phonological awareness skills, *n* = 22; (3) poor auditory processing with poor attention and memory but good language skills, *n* = 15; and (4) poor auditory processing and attention with good memory skills, *n* = 18.

**Conclusion:**

The cluster analysis identified distinct subgroups of children. These subgroups display the variation in areas of difficulty observed across different studies in the literature (e.g., not every child with APD has an attention deficit), highlighting the heterogeneous nature of APD and the need to assess a range of skills in children with suspected APD. It would be valuable for future studies to independently verify these subgroups and to determine whether interventions can be optimized based on these subgroups.

## Introduction

Some school-aged children appear to not hear well in difficult listening situations such as the classroom, in the absence of a hearing loss based on pure tone audiometry (Purdy et al., 2018). These children are often described as having problems hearing in noise, needing to have instructions repeated, being unable to follow verbal instructions and having generally poor listening skills (Chermak et al., 2002). Some of these children also show co-existing reading difficulties and/or attention deficits (Richardson et al., 2004; Sharma et al., 2009; Tomlin et al., 2015). These children are initially tested for hearing loss and in the absence of any audiometric hearing loss they should be referred for auditory processing assessment (Jerger and Musiek, 2000). Clinical practice varies widely, however, despite considerable efforts internationally to develop auditory processing assessment and treatment guidelines (Iliadou et al., 2018).

In children diagnosed with APD, there is impaired processing of auditory information that is not consistent with their hearing thresholds (Moore et al., 2013). Auditory processing includes the ability of the auditory system to localize, discriminate, recognize auditory patterns, and discriminate temporal aspects of sounds (including but not limited to temporal resolution, masking, integration and sequencing) (American Speech and Hearing Association [ASHA], 1996; Jerger and Musiek, 2000). A significant deficit in any of these auditory skills is indicative of APD (American Speech and Hearing Association [ASHA], 1996). Thus to diagnose any child with APD, many established guidelines (American Speech and Hearing Association [ASHA], 1996; Jerger and Musiek, 2000; Wilson, 2018) recommend a test battery that evaluates multiple auditory processing skills.

Clinicians working with children with suspected APD face three important challenges. One is that auditory processing is not a unitary skill and therefore cannot be assessed with one test (Jerger and Musiek, 2000; Wilson, 2018), hence clinicians need to access a range of tests that have age-dependent norms and demonstrated reliability, test efficiency and validity (Musiek et al., 2010; Emanuel et al., 2011; Wilson, 2018; Keith et al., 2019). For example, commonly used tests such as the FPT (Musiek, 1994) and the DDT (Musiek, 1983) have age-related norms while the Random Gap Detection Test (RGDT) has a screening pass level that is applied to all school aged children (Sharma et al., 2006; Kelly, 2007). A second challenge is that children with APD can have co-existing language, attention, and/or reading disorders (Sharma et al., 2009; Wilson, 2018) that may affect test results and/or management choices. A third challenge stems from the need for efficient, clinically feasible diagnostic protocols that can capture APD in children who are heterogeneous and that assist the children and their families in receiving appropriate management that includes appropriate evidence-based treatments (Wilson, 2018). The current research attempts to address the second and third challenges by determining whether there are identifiable subprofiles of children who are suspected to have APD with other potential co-existing disorders, since such subprofiles may help guide management. The research aim is to determine whether cluster analysis identifies distinct subgroups of children, which would help researchers and clinicians to better understand the range of challenges that children with APD present with, and could guide recommendations to parents and clinicians regarding appropriate clinical referral pathways. There have been attempts to define subgroups of children with APD in the past (Bellis and Ferre, 1999), recognizing the potential values of this approach for clarifying referral pathways and planning treatment, but to our knowledge the current study is the first that uses cluster analysis to define subgroups.

There is ample evidence that children with auditory processing deficits can display reading and language deficits, but typically this is not the case for all children (Jerger and Musiek, 2000; Ramus, 2003; Bishop, 2007; Sharma et al., 2009; Leppänen et al., 2010; Hämäläinen et al., 2013; Halliday et al., 2017; Mealings and Cameron, 2019). A causal relationship between auditory deficits and poor reading and/or language skills has been proposed, or at least it has been suggested that these share some common underlying neurodevelopmental etiology (Leppänen et al., 2010; Moore et al., 2013; Halliday et al., 2017). This is difficult to prove, however, and there is no empirical evidence that confirms this. A theoretical framework has been proposed (Ramus, 2003; Goswami, 2011; Halliday et al., 2017) that attempts to explain why auditory processing and reading disorders are associated (Sharma et al., 2006; Leppänen et al., 2010; Hämäläinen et al., 2013) but there is no agreement on the “nature or magnitude of the link” between auditory processing and reading disorders (Ramus, 2003). There are also reports that children with auditory processing deficits have cognitive (attention and/or working memory) difficulties that account, at least in part, for their poor performance on auditory processing tests (Moore et al., 2013). This is also not straightforward as some children with APD do not have attention and memory deficits (Sharma et al., 2009, 2014a; Tomlin et al., 2015).

Links between auditory, cognitive, reading, and language abilities of children with APD are still not fully understood. It is recommended that children with suspected APD are assessed using a wide range of measures that encompass all these domains (American Speech Language Hearing Association [ASHA], 2005). In the current study auditory, cognitive, reading, and language abilities of children with suspected APD were assessed and cluster analysis was used to determine whether the results revealed distinct subgroups of children. The subgrouping was then tested by comparing the groups across a range of related measures not included in the cluster analysis to determine where there were significant differences in performance.

## Methodology

### Participants

The University of Auckland Human Research Participants’ Ethics Committee approved this study. Written informed consent to participate in this study was provided by the participants’ legal guardian/next of kin. Ninety children aged 7–12.8 years old (*Mean* = 9.8 years ± 1.5) with listening concerns participated: 58 males with an average age of 9.8 years ± 1.6 and 32 females with an average age of 9.7 years ± 1.5. Children were referred to the study by speech language pathologists, teachers, educational psychologists, and audiologists. Most came to the research with suspected APD and reports of other reading and language concerns, making this a potentially heterogeneous group of participants. A subset of these children was reported on previously (Sharma et al., 2009, 2019; Gilley et al., 2016).

### Methods

Children were tested individually in a sound-treated laboratory booth over two sessions of about 3 h each with multiple breaks. Pure-tone audiometry and behavioral auditory processing tests were administered using a GSI clinical audiometer and TDH-39 earphones. Test materials were presented at 60 dB HL using a CD player (Bass XPander, P882). All children were administered hearing, auditory processing (behavioral and electrophysiological), language, cognitive, and reading assessments.

Inclusion criteria included normal peripheral hearing and a standard score of 80 or more on the TONI (Brown et al., 1990). Parents were invited to report on their children’s perceived listening difficulties by completing the Children’s Auditory Performance Scale (CHAPS) questionnaire (Smoski et al., 1998), which rates the children’s difficulties compared to classroom peers (a score a “0” indicates equivalent performance to peers) (Sharma et al., 2009). Smoski et al. (1998) proposed a normative cut-off of −11 for the overall CHAPS score, with scores lower than this indicating significant listening difficulties. In total 83 parents (92% of participants) returned the CHAPS questionnaire.

All participants had normal hearing sensitivity. Pure tone thresholds were 15 dB HL or better at octave frequencies from 250 to 8000 Hz. All children had Type A tympanograms, measured using a 226-Hz probe tone (Jerger, 1970) and ipsilateral 1000-Hz acoustic reflex thresholds less than 100 dB HL (Silman and Gelfand, 1981) consistent with normal middle ear function. For all children otoacoustic emissions (OAE) strength was within the normal range based on the pass/refer criteria in the TEOAE protocols of the Scout Sport System (Bio-Logic Systems Corp^®^) (Hall, 2000).

Children were evaluated on multiple measures after completing the peripheral hearing assessments. The tasks included in the cluster analysis are the ones where published age-specific norms were available. Details of the stimuli, procedure and scoring are provided in Table 1.

**TABLE 1:** The details of the all tests that included auditory processing, reading, language, attention, memory in the current study.

	**Tests**	**Procedure**
Auditory processing	Frequency Pattern Test (FPT)	*Stimuli*: triplet tones presented in a sequence of high and low frequencies. The high frequency had a frequency of 1100 Hz, and the low tone had a frequency of 880 Hz (Musiek, 1994). The tones were presented through an Audiometer that received the input from a CD player. The tones were 150 ms long with an interstimulus interval of 80 ms. There were six possible sequences, HLL, HLH, HHL, LHH, LHL, LLH.
		*Procedure*: the participants had to verbally identify the sequence.
		*Response and scoring*: the verbal response of the participants were marked, with each correct response yielding 1 point. The score out of 15 was calculated to a percentage score and converted to *z* scores.
	Dichotic Digit Test (DDT)	*Stimuli*: in this task two pairs of double digits (four digits) are presented to the two ears simultaneously and the listener has to verbally report back the four digits.
		*Procedure:* the participants repeat the numbers they hear irrespective of the order (free recall). Twenty digits were presented in each ear.
		*Response and scoring*: each correctly identified digit is allocated a score of 1 and percentage correct for each ear is calculated out of maximum presented digits (20 for each ear) and converted to *z* scores.
	RGDT	*Stimuli*: in this task two clicks are presented to both ears with varying gap and the listener has to verbally report back whether they heard 1 click (no gap) or 2 clicks (gaps of varying duration).
		*Procedure*: the participants state they hear 1 or 2 clicks. The gaps include 0, 2, 5, 10, 15, and 20 ms.
		*Response and scoring*: the shortest gap identified is noted as the threshold.
	MLD	*Stimuli*: 500-Hz tone masking level difference task is a measure of the improvement in detectability of a tone when presented in noise that is phase-shifted between the two ears.
		*Procedure*: the listener has to identify the presence of tone within noise. Detection of the tone improves for the phase-shifted binaural listening condition compared to the in-phase condition.
		*Response and scoring*: the MLD is the 500-Hz threshold difference when the S_Π_ N_0_ (tone presented to the two ears in opposite phase, noise presented in the same phase) and S_0_N_0_ (tone and noise presented to the two ears in the same phase) conditions are compared.
	AB words	*Stimuli*: in this task, monosyllabic words (Consonant-vowel-consonant) are presented to each ear and the listener has to verbally report back each word.
		*Procedure*: the participants repeat the words they hear. Ten words were presented in each ear in quiet and modified by 65% compression and 0.3s reverberation.
		*Response and scoring*: each correctly identified word is allocated 10% and each consonant within each word is allocated a score of 3%. Scores were separately determined for quiet and modified for right and left ear.
	CAEPs	*Stimuli*: /da/ natural token 158 ms long stimulus spoken by Australian female, are presented to both ears via insert earphones in quiet at 70 dB SPL and +3 dB SNR.
		*Procedure*: the listener does not need to respond and was asked to watch a muted movie of their choice. CAEPs were collected from the vertex (Cz) and frontal (Fz) non-inverting electrodes, with right and left mastoid as inverting electrode.
		*Response and scoring*: P1-N250 peaks were identified. P1 is a biggest positive peak at about 100 ms after the onset and N250 is the biggest negative peak that occurs at about 250 ms after the onset of the sound. Amplitude and latency for P1-N250 were identified.
Reading	Castles and Coltheart	*Stimuli*: the Castles’ Word/Non-word Test (Castles and Coltheart, 1993) was used to measure word reading for regular words (pronounced in accordance with letter-sound rules, e.g., plant), irregular words (pronunciation violates letter-sound rules, e.g., yacht), and non-words (e.g., phot).
		*Procedure*: the words from the three lists are presented in a random order.
		*Response and scoring*: the participant receives a score of 1 for each word read correctly. There were 30 words in each list.
Phonological processing	Queensland Inventory of Literacy (QUIL)	*Stimuli*: seven QUIL subtasks were utilized for testing phonological processing; non-word spelling, syllable identification and segmentation, rhyming, spoonerisms, phoneme detection; phoneme manipulation.
		*Procedure*: non-word spelling; syllable identification (to determine the number of syllables in a given word); syllable segmentation; rhyming (judgment of auditory word pairs, such as “shell and bell”, or “bout and bait”); spoonerisms (swapping the first sounds from a pair of spoken words to make new words such as “felt and make” swapped to make “melt and fake”); phoneme detection (identifying the odd word out, which may be different because of first/second or third sound); and phoneme segmentation and manipulation (identifying and removing a sound from a given word and then saying the new word; e.g., “frog” without *r* is “fog”).
		*Response and scoring*: the participant receives a score of 1 for each item within each task completed correctly and converted to scaled scores.
Non-verbal intelligence	TONI	*Stimulus*: the TONI-3 measures a child’s reasoning ability with minimal language influence. It is a norm-referenced measure of cognitive ability that assesses intelligence aptitude, abstract thinking, and problem solving for 6–89 year olds.
		*Procedure*: for each item, the child selected one of six options to complete a matrix pattern that incorporated one or more features, such as shape, direction, position, size, and movement.
		*Response and scoring*: the correct identification of pattern led to total scores that are then converted to standard scores.
Memory	Digit Span Test	*Stimulus*: auditory memory was assessed using the CELF-4 forward and backward digits tasks.
		*Procedure*: the participant recalls a series of numbers in either forward or reverse order immediately after hearing them. The numbers are spoken slowly in a monotone voice and the child is asked to repeat them. The length of the series keeps increasing until the child can no longer repeat the series correctly in the appropriate sequence. Each sequence is presented in pairs.
		*Response and scoring*: score of 1 is given for every correct sequence till the participant made an error for a pair of sequence. The score is determined independently for forward sequences and reverse order sequences. These were then converted to scaled scores.
Sustained attention	Visual and Auditory Continuous Performance Test	*Stimulus*: children’s attention was tested using the IVA (Integrated Visual and Auditory Continuous Performance Test) presented on a laptop computer, which assesses performance in a combined visual and auditory task (Sandford and Turner, 1995).
		*Procedure*: numbers 1 and 2 are seen and heard in pseudorandom order. The task includes 500 trials and takes approximately 15 min to complete. Feedback is provided only during practice trials. The child was instructed to click the mouse whenever the number “1” was seen or heard, and to ignore the number “2”.
		*Response and scoring*: the task is undertaken for about 15 min and based on the number of correctly identified responses in both auditory and visual modality, the sustained attention standard score is calculated independently for auditory and visual modality.
Language	CELF-4	*Stimulus*: the Clinical Evaluation of Language Fundamentals 4th edition (CELF-4; Semel et al., 2003) was used to assess children’s receptive and core language scores.
		*Procedure*: the Receptive Language score is a cumulative score from the subtests Concepts and Following Directions, Receptive Word Classes (semantic relationships), and Sentence Structure. Expressive Language is a cumulative score from subtests such as recalling sentences, formulated sentences and word classes-expressive.
		*Response and scoring*: the correct responses in all subtests are added and converted to scaled scores.
	CASL	*Stimuli*: comprehensive Assessment of Spoken Language (CASL) is an orally presented language assessment battery for ages 3–21 years (Carrow-Woolfolk, 1999).
		*Procedure*: two tasks were included Auditory Comprehension and Supralinguistics. Auditory Comprehension included two subtests paragraph comprehension and synonym while Supralinguistics included subtests non-literal (explaining sentences) and inference drawing (determining meaning).
		*Response and scoring*: the correct responses in all subtests are added and converted to scaled scores.

The auditory processing measures were the FPT (Musiek, 1994) and DDT (Musiek, 1983). Cognitive measures were memory (Comprehensive Evaluation of Language Fundamentals, CELF-4, digit span forward and backward) (Semel et al., 2003) and sustained attention [Integrated Visual and Auditory (IVA) Continuous Performance Test] (Sandford and Turner, 2000). Language measures were Receptive and Core Language standard scores from the CELF-4, and Auditory Comprehension and Supralinguistics standard scores from the Comprehensive Assessment of Spoken Language (CASL), as these all rely on auditory perception (Carrow-Woolfolk, 1999). The reading task included was word reading measured using the Castle and Coltheart’s word lists (Castles and Coltheart, 1993). Phonological processing was measured using the Queensland Inventory of Literacy (QUIL) (Dodd et al., 1996). Only those QUIL tasks specifically linked to auditory perception were included (syllable identification, segmentation, rhyming, spoonerisms, phoneme detection, phoneme manipulation). Non-word spelling and visual rhyme subtests were not included in the cluster analysis. All analyses were undertaken using Statistica 10.0.

### Data Reduction: Correlation and Factor Analysis

The entire dataset included 23 variables. Pearson correlation analyses were undertaken to remove highly correlated variables. This is important step as strongly correlated variables represent potentially the same measure and may receive higher weighting during cluster analysis. Both correlation and factor analysis were undertaken to avoid this (Chiarello et al., 2012). Variables with strong correlations (*r* ≥ 0.70) were not placed in the cluster analysis (Taylor, 1990). Following the correlational analysis, exploratory factor analysis was used to further reduce the number of variables.

### Cluster Analysis

Before undertaking the cluster analysis, the selected variables were standardized to control for unequal scaling of the data (Clatworthy et al., 2005). The standardization transforms all values (regardless of their distributions and original units of measurement) to compatible units from a distribution with a mean of 0 and a standard deviation of 1. This transformation makes the distributions of values easy to compare across variables and independent of the units of measurements. A hierarchical cluster analysis using Ward’s method was performed on the data to determine how many clusters are appropriate for the final selected variables (Clatworthy et al., 2005; Chiarello et al., 2012). Following this, a k-means cluster analysis was performed on the data to determine the membership of the individual cases into the clusters. Once group membership was determined, a discriminant function analysis was undertaken to confirm predicted membership.

### Inferential Statistics

The stability of the clustering was determined by comparing groups on the variables that were not used in the cluster analysis to evaluate generalizability of the clusters (Chiarello et al., 2012). The group comparisons included gender distribution, paragraph reading [Wheldall Accuracy of Reading Passages (WARP)] (Madelaine and Wheldall, 1998), non-word spelling (QUIL subtest), CELF-4 Expressive language (Semel et al., 2003), RGDT (Keith, 2000), Masking Level Differences (MLD) (Sweetow and Reddell, 1978; Jerger et al., 1984), word recognition scores (AB words in quiet and in noise with 65% compression and 0.3s reverberation of words) (Sharma et al., 2009), and speech-evoked cortical auditory evoked potential (CAEP) latencies and amplitudes. The procedure for recording CAEPs to /da/ in quiet and in noise (at 3 dB signal-to-noise ratio, SNR) is described elsewhere (Sharma et al., 2014b). All comparisons were performed with age as a covariate and results were adjusted for multiple testing using Bonferroni correction.

## Results

### Data Reduction: Correlation and Factor Analysis

FPT and DDT scores for right and left ears were significantly correlated (*r* = 0.86, *p* < 0.001 and *r* = 0.070, *p* < 0.001, respectively) and therefore, only FPT and DDT right ear scores were included in the cluster analysis. Castle and Coltheart regular word and irregular word scores were also highly correlated (*r* = 0.78, *p* < 0.001) so only irregular words were included. Non-word scores on the Castle and Coltheart test and QUIL were correlated (*r* = 0.72, *p* < 0.001); the Castle and Coltheart non-word task was included in the cluster analysis and the QUIL subtests were examined separately.

Tables 2A,B provide Pearson’s correlational results for QUIL and language tasks respectively. Scores for the QUIL subtests were weakly or modestly correlated with each other (*r* values in the range 0.28–0.55). All QUIL measures were therefore included in the next stage of data reduction using factor analysis (Taylor, 1990) (Table 2A).

**TABLE 2:** Pearson correlations.

**(A)**								
		**1**	**2**	**3**	**4**	**5**	**6**	**7**
QUIL	Non-word reading							
Syllable identification	0.26^ns^							
Syllable Segmentation	0.36^∗∗^	0.19^ns^						
Spoken rhyme	0.55^∗∗^	0.28^∗^	0.16^ns^					
Spoonerism	0.46^∗∗^	0.43^∗∗^	0.43^∗^	0.37^∗∗^				
Phoneme detection	0.44^∗∗^	0.33^∗^	0.44^∗∗^	0.34^∗^	0.48^∗∗^			
Phoneme segmentation	0.24^ns^	0.21^ns^	0.15^ns^	0.25^ns^	0.22^ns^	0.21^ns^		
Phoneme manipulation	0.50^∗∗^	0.34^∗^	0.34^∗^	0.49^∗∗^	0.52^∗∗^	0.50^∗∗^	0.26^ns^	

**(B)**								
	**Core**	**Auditory Comprehension**	**Supralinguistics**

CELF: Receptive			
CELF: Core	0.69^∗∗^		
CASL: Auditory Comprehension	0.52^∗∗^	0.52^∗∗^	
CASL: Supralinguistics	0.54^∗∗^	0.63^∗∗^	0.52^∗∗^

***(A)** Word reading and phonological awareness tasks as measured on QUIL across all children (*N* = 90). **(B)** Language subtasks as measured on CELF-4 and CASL across all children (*N* = 90). ^∗∗^*p* < 0.0001, ^∗^*p* < 0.001, ns = *p* > 0.01.*

Performance on the memory tasks (digit span forward and backward) were significantly correlated but the correlation was weak (*p* = 0.001, *r* = 0.35); both measures were included in the cluster analysis. Auditory and visual sustained attention were strongly correlated (*p* < 0.001, *r* = 0.74) and therefore, only auditory attention scores were included. Language scores were not highly correlated (*r* values 0.52–0.69) (Table 2B), therefore, all languages scores (Receptive, Core, Auditory Comprehension, Supralinguistics) were included in the next stage of data reduction using factor analysis.

Of the now 18 tasks included based on the correlation analysis, there were six measures of phonological processing and four language measures. From the six measures of phonological processing, (unrotated) principal component analysis identified only one factor with an eigenvalue greater than 1, explaining 48.6% of the variance. This included all six items with loadings greater than 0.50 (Table 3). Individual principal component scores were therefore included in the cluster analysis. Similarly, when the unrotated principal component analysis was undertaken for the four language measures, only one principal component was extracted that explained 68.0% of the variance. All four items within the component had loadings greater than 0.77 and hence the principal component scores were used for the cluster analysis.

**TABLE 3:** Factor loadings and communalities based on a (unrotated) principal components analysis for six items from the **(A)** QUIL subtests and **(B)** language measures from CELF-IV and CASL (*N* = 90).

**Items**	**Factor 1^∗^**
**(A)**	
Syllable identification	0.60
Syllable segmentation	0.60
Spoken rhyme	0.62
Spoonerism	0.79
Phoneme detection	0.75
Phoneme Manipulation	0.78
**(B)**	
CELF: Receptive	0.84
CELF: Core	0.87
CASL: Auditory Comprehension	0.77
CASL: Supralinguistics	0.82

*^∗^The factor scores were multiplied by −1 to facilitate interpretation of results.*

### Classification Analyses: Predictors of Cluster Membership

For the next stage of analysis, the now remaining 10 measures were standardized. The tasks included were auditory processing (FPT, DDT), reading (irregular, non-word), language (one principal component derived from Receptive, Core, Auditory Comprehension, Supralinguistics), TONI, phonological processing (one principal component derived from syllable identification and segmentation, spoken rhyme, spoonerism, phoneme detection, and manipulation), sustained auditory attention, and both memory measures (digit span forward and backward). All the values for the factors were within two standard deviations of the mean and therefore, no outliers were identified.

**FIGURE 1:**
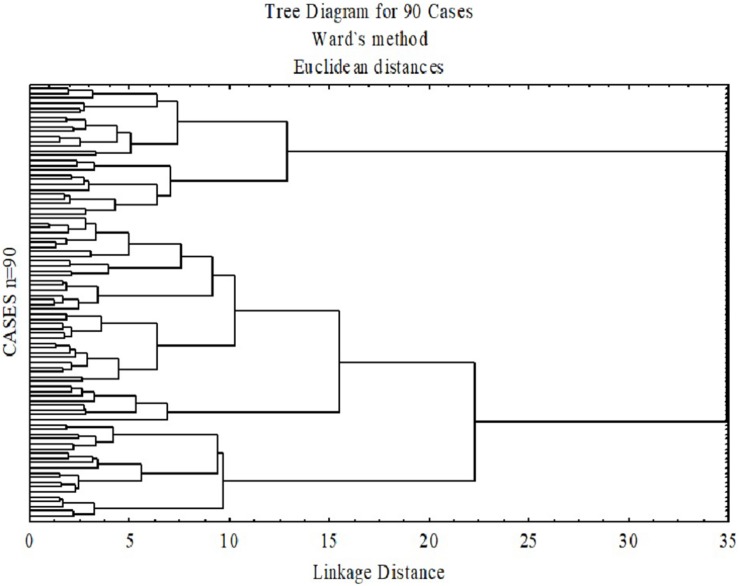
Dendrogram based on Wald’s minimum-variance hierarchical clustering method. The 90 participants were clustered into a single final group. At each generation of clusters, samples were merged into larger clusters to minimize the within cluster sum of squares.

**FIGURE 2:**
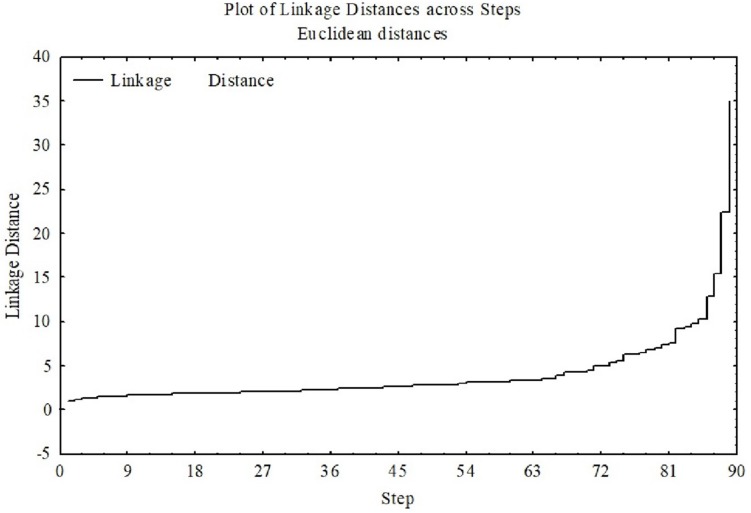
Linkage distance with a clear plateau means that many clusters were formed at essentially the same linkage distance. The higher the linkage distance (*y*-axis), the more dissimilar the groups are.

**FIGURE 3:**
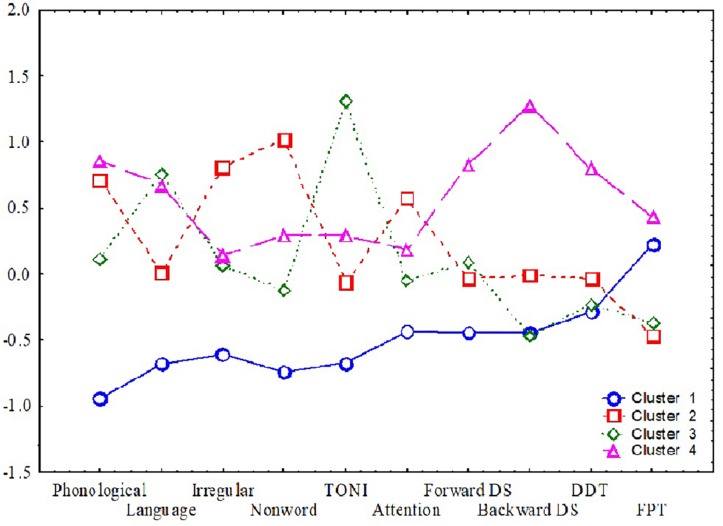
Means for the four clusters. The *y*-axis shows the means (of standardized scores such that +1 is one standard deviation better than the average sample score) and the *x*-axis shows the 10 variables used to determine the clusters.

The hierarchical cluster analysis, as seen in Figure 1, suggested a four-cluster solution appropriate for the final 10 selected variables. Using the plot of linkage distances, Figure 2 shows a plateau, thus a large number of clusters were at the same linkage distance. A four-cluster solution was determined at a point where the plateau ended between linkage distances of 10–15. The final grouping of cases into four clusters was determined after three iterations of the k-means algorithm, using equally spaced centers. Figure 3 shows the means of the 10 standardized variables in each of the four clusters.

The discriminant analysis and the cluster analysis showed very similar membership of the cases. The accuracy was 97% for group one, 95% for group 2, 93% for group 3, and 89% for group 4. Box’s *M* test (*p* = 0.134) was not significant, indicating that the assumption of homoscedasticity is justified. A significant Wilks lambda (Λ = 0.072, *p* < 0.001) shows a good difference in the mean scores between the four clusters. Table 4 shows the demographics and performance on auditory processing, reading, language and cognitive skills of children across the four clusters.

**TABLE 4:** **(A)** Demographics and means (and standard deviation) of participants within each cluster on all skills; **(B)** means (and standard deviation) of participants within each cluster on phonological processing and language.

**Variable**	**Total *N* = 90**	**Cluster 1, *N* = 35 poor reading and language**	**Cluster 2, *N* = 22 good word reading skills and attention, poor FPT**	**Cluster 3, *N* = 15 good non-verbal IQ (TONI) and language skills, poor AP**	**Cluster 4, *N* = 18 good memory (digit span), language skills and AP**

**(A)**					
Age	9.7 (1.5)	9.5 (1.4)	10.4 (1.4)	10.2 (1.6)	9.3 (1.7)
Gender (F, M)	38, 52	13, 22	7, 15	4, 11	8, 10
Irregular word reading	17.4 (6.7)	13.5 (6.3)	**22.6 (3.5)**	18.3 (6.9)	17.6 (6.1)
Non-word reading	16.5 (8.2)	10.1 (6.4)	**24.3 (3.6)**	17.9 (5.2)	18.3 (7.9)
Forward digit span	6.5 (2.4)	5.4 (2.0)	6.4 (2.3)	6.7 (2.3)	** 8.5 (2.4)**
Backward digit span	8.5 (2.8)	7.3 (2.0)	8.5 (2.1)	7.2 (2.2)	**12.1 (2.3) **
Auditory attention	77.0 (33.7)	62.4 (37.0)	** 96.2 (20.0)**	75.3 (29.0)	83.2 (32.5)
TONI	99.8 (13.1)	91.0 (7.3)	99.1 (8.2)	**117.0 (13.6) **	103.7 (11.2)
DDT raw scores^#^	78.1 (13.9)	72.4 (15.4)	80.2 (9.8)	77.3 (14.1)	87.3 (9.2)
DDT	0.0 (1.0)	−0.3(1.1)	−0.04(0.7)	−0.2(1.1)	** 0.8 (0.4)**
FPT raw scores^#^	43.8 (27.9)	28.9 (9.3)	62.2 (24.7)	49.3 (27.8)	45.9 (31.7)
FPT	0.0 (1.0)	0.2 (1.1)	−0.5(0.6)	−0.4(0.8)	** 0.4 (1.1)**

**(B)**					

Syllable identification	7.5 (3.6)	5.6 (3.2)	8.7 (3.8)	7.8 (3.0)	** 9.7 (2.6)**
Spoken rhyme	6.1 (3.3)	3.7 (1.4)	8.1 (3.0)	6.3 (3.2)	** 8.3 (3.2)**
Spoonerism	7.2 (3.8)	4.3 (2.4)	9.2 (3.1)	7.6 (3.8)	**10.0 (3.4)**
Phoneme detection	8.5 (3.7)	6.4 (3.3)	**10.6 (3.0)**	8.3 (3.9)	10.3 (3.1)
Phoneme manipulation	7.4 (3.3)	4.8 (2.8)	9.6 (2.1)	7.7 (2.7)	** 9.7 (2.3)**
Receptive	87.6 (14.3)	79.5 (11.9)	89.5 (11.3)	** 94.9 (13.9)**	** 94.9 (15.3)**
Core	83.7 (13.2)	74.4 (9.7)	84.1 (10.5)	** 93.7 (10.2)**	93.0 (12.2)
Auditory Comprehension	98.7 (12.6)	92.7 (11.9)	98.6 (9.6)	**106.7 (12.7) **	103.9 (11.7)
Supralinguistics	93.8 (11.6)	88.31 (12.3)	92.4 (10.3)	100.7 (9.9)	**100.7 (6.4)**

***(A)** Bold numbers indicate the cluster with the highest average performance on a given skill. All scores are standardized scores unless otherwise stated. ^#^DDT and FPT raw scores are percent correct irrespective of age. **(B)** Bold numbers indicate the cluster with the highest average performance on a given skill. All scores are standardized scores unless otherwise stated.*

**FIGURE 4:**
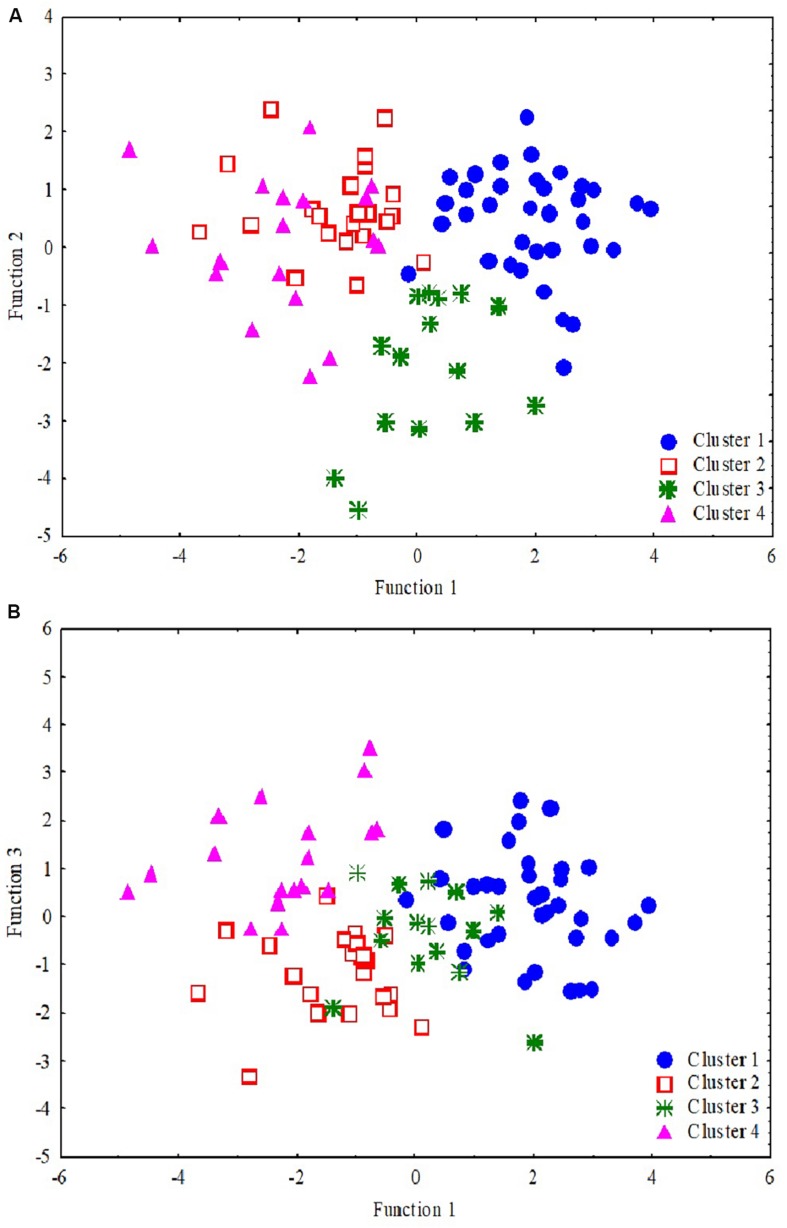
Scatterplot of the discriminant functions. Each data point represents a single participant where cluster 1 (blue dots, *n* = 35), cluster 2 (red empty square, *n* = 22), cluster 3 (green star, *n* = 15), and cluster 4 (pink triangle, *n* = 18). The two plots depict clustering and separating of four clusters using three factors (of the 10 variables). While **(A)** shows that the clusters 1 and 3 are separated clearly by functions 1 and 2, **(B)** shows that clusters 2 and 4 separate very clearly by functions 1 and 3.

Four variables that provided a three-function solution had much higher discriminant function coefficients and hence were more relevant in determining cluster membership. These variables were phonological processing, digit span backward (function 1), TONI (function 2), and non-word reading (with backward digit, function 3) (Λ = 0.1, *p* ≤ 0.004 for all) (Supplementary Table 1). Figures 4A,B shows the scatterplot of cases for the four clusters across the three functions.

### Inferential Statistics

Table 5 shows how the four clusters differ on the additional auditory processing and language tasks that were not included in the cluster analysis. The four clusters of children did not differ significantly based on any of the auditory processing measures including CAEPs (Table 5). There were significant performance differences between the four clusters, however, for several reading, phonological, and language measures (WARP paragraph reading, non-word spelling, visual rhyme, expressive language) (Table 5).

**TABLE 5:** Clusters in predicting reading, auditory processing performance, only significant results on ANOVA are shown.

**Variables**	**Cluster 1 mean (SD) *N* = 35**	**Cluster 2 mean (SD) *N* = 22**	**Cluster 3 mean (SD) *N*** =** 15**	**Cluster 4 mean (SD) *N* = 18**	**Test of significance**	**Tukey *post hoc***
CHAPS^#@^	−11.2(4.6)	−11.6 (5.3) *N* = 20	−7.0 (4.4) *N* = 11	−6.9 (8.8) *N* = 17	*F*(3,78) = 3.28, *p* = 0.20^$^	ns^∗^
WARP	60.6 (40.4)	116.6 (27.9)	81.6 (49.7)	84.9 (48.7)	*F*(3,85) = 7.41, *p* = 0.002^$^	Cluster 1 vs. 2: *p* = 0.002
Non-word spelling (QUIL)	3.5 (1.2)	6.0 (2.3)	4.0 (1.7)	7.4 (2.9)	*F*(3,85) = 21.42, *p* < 0.001	Cluster 1 vs. 2: *p* = 0.0001; Cluster 1 vs. 4: *p* = 0.0008 Cluster 2 vs. 3: *p* = 0.072; Cluster 3 vs. 4: *p* = 0.002
Visual rhyme (QUIL)	5.3 (2.8)	7.0 (3.8)	5.0 (2.8)	4.0 (1.7)	*F*(3,85) = 12.75, *p* < 0.001	Cluster 1 vs. 4: *p* = 0.0008; Cluster 2 vs. 4: *p* = 0.032; Cluster 3 vs. 4: *p* = 0.002
Expressive language (CELF-IV)	75.9 (11.0)	85.4 (11.5)	92.0 (12.4)	93.3 (12.7)	*F*(3,85) = 11.62, *p* < 0.001	Cluster 1 vs. 2: *p* = 0.16; Cluster 1 vs. 3: *p* = 0.002; Cluster 1 vs. 4: *p* = 0.002;
RGDT	16.8 (14.5)	11.3 (10.3)	10.7 (10.3)	14.6 (14.0)	*F*(3,85) = 0.45, *p* = 0.715^$^	ns^∗^
MLD	10.5 (4.3)	11.6 (2.5)	10.2 (2.1)	11.1 (3.4)	*F*(3,85) = 0.61, *p* = 0.607^$^	ns^∗^
AB words 65%, 0.3s (*R*)	76.0 (10.2)	75.0 (9.3)	74.5 (8.3)	75.9 (6.3)	*F*(3,85) = 0.03, *p* = 0.994	ns^∗^
Hint sentences 65%, 0.3s (R)	57.5 (17.6)	68.7 (21.1)	66.3 (15.6)	73.4 (20.8)	*F*(3,85) = 4.48, *p* = 0.048	ns^∗^
CAEPS in Q and N 250 amp					*F*(3,85) = 1.99, *p* = 0.121	ns^∗^
CAEPS in Q and N 250 latency					*F*(3,85) = 1.99, *p* = 0.121	ns^∗^
CAEPS in Q and P1 amp					*F*(3,85) = 1.30, *p* = 0.279	ns^∗^
CAEPS in Q and P1 amp					*F*(3,85) = 1.30, *p* = 0.279	ns^∗^
CAEPS in Q and P1 latency					*F*(3,85) = 0.75, *p* = 0.526	ns^∗^

*For some variables, univariate analysis was conducted (shown with *^$^*). The variables in bold are distinct across the clusters. Significance is adjusted for multiple testing. ^∗^*p* > 0.02 is suppressed. #CHAPS = Children’s Auditory Performance Scale only completed by 83 caregivers. @ Not all parents completed the CHAPS (*n* = 83) and more negative implies more difficulty. $ Univariate analysis.*

### Interpretation of Clusters

Four clusters emerged based on ten tasks included in the cluster analysis. To determine differences between clusters, each skill was scaled against the mean to determine the proportion of children with relatively poor results for the different areas included in the cluster analysis.

Cluster 1 included 35 children who showed overall poor scores on reading, language, and cognitive measures. Dichotic scores were also impacted relatively more in this group compared to other clusters. This cluster of children appear to have global deficits across all domains. All children had scores more than 1 SD below the overall mean (*N* = 90) for more than one measure (Tables 4A,B). One quarter of the children in this cluster had TONI standard scores of 80–85 (a standard score of 80 was the lower limit for study inclusion), and 63% had scores more than 1 SD below the mean for sustained auditory attention. About half of the Cluster 1 parents (51%) reported that their children had significant listening difficulties based on the CHAPS scoring criterion proposed by Smoski et al. (1998) (overall score < −11). Children within this cluster also showed performance 2 SD below the mean on FPT (*n* = 12, 34%), DDT (*n* = 3, 9%) or both (*n* = 19, 54%). Pearson’s partial correlations within the cluster exploring associations between cognitive, reading, and language skills, and DDT and FPT auditory processing measures (with age as covariate) showed no significant associations (with Bonferroni adjustments).

Cluster 2 included 22 children with good reading and good phonological processing skills. Only one child had a TONI score more than 1 SD below the mean and this child had high reading and phonological skills. Another child had a score more than 2 SD below the mean for the digit span backward test but had average language, reading, and phonological processing scores. Auditory processing skills measured using the FPT and DDT showed that 27% of this group only had FPT scores that were more than 2 SD below the mean, 18% only had DDT scores more than 2 SD below the mean, while 27% had poor performance on both the FPT and DDT. This cluster includes children with auditory processing difficulties in the presence of relatively good reading and phonological processing skills and, like Cluster 1, the CHAPS showed that half of the children in this cluster had parent-reported listening difficulties (overall score < −11) (Smoski et al., 1998). This cluster showed a moderate and significant partial correlation (age as covariate) between non-word reading and paragraph reading (*r* = 0.58, *p* = 0.048).

Cluster 3 included 15 children with relatively high non-verbal IQ scores, and good phonological processing and word reading. Thirty percent of children in this cluster had FPT scores that were more than 2 SD below the mean, while 13% only had DDT scores that were more than 2 SD below the mean, and 40% of children had scores 2 SD below the mean for both FPT and DDT. Four children with scores more than 2 SD below the mean for FPT and DDT also showed scores 1 SD below the mean on sustained auditory attention and working memory (backward digit span) tasks. In general, this cluster had good TONI and language skills with poor auditory processing and poor attention and memory and about 27% of parents (3/11 who completed the questionnaire) reported listening difficulties based on the CHAPS criterion. For this cluster, the DDT showed a significant partial correlation (age as covariate) with digit span forward scores (*r* = 0.69, *p* = 0.048).

Cluster 4 included 18 children mostly with at least average scores on all tasks other than FPT. Three children who showed DDT deficits with scores more than 2 SD below the norm also showed difficulties with the FPT. Forty four percent of children had difficulties only on FPT and seven of these also had poor sustained attention deficits. This cluster represents children with good memory, word reading, and language skills, combined with poor FPT scores and sustained attention. For cluster 4, 35% of the parents (6/17) reported listening difficulties based on responses to the CHAPS questionnaire. Although not significant, a trend was observed for an association between non-word reading and phonological processing (*r* = 0.62, *p* = 0.064) and between Core Language and paragraph reading (WARP, *r* = 0.62, *p* = 0.064).

## Discussion

Children with suspected APDs have been reported to differ from control group children without auditory difficulties on measures of attention, memory, reading and/or language skills. Comorbidity of APDs with other neurodevelopmental conditions is a norm rather than an exception (Sharma et al., 2009; Musiek et al., 2010; Tomlin et al., 2015); the proportion of children with co-occurring conditions varies across studies but is typically about 40–50% (Sharma et al., 2009; Ferguson et al., 2011). Variations are likely to reflect sampling and test protocol differences across studies. These studies have largely been cross-sectional and have used simple group comparisons, analysis of variance, and regression and correlation analyses to demonstrate links between different domains of neurodevelopmental difficulties.

A cluster analysis is unsupervised, in other words, it does not employ any *a priori* restrictions. Consequently, cluster analysis offers an advantage over other approaches in determining distinct groups based on dominant features or common skills (Clatworthy et al., 2005; Chiarello et al., 2012). The current analyses provide evidence for the validity of four clusters of children amongst the 90 children referred to the study with suspected APDs. These clusters differentiate largely based on backward digit span, phonological processing, and non-verbal intelligence with smaller contributions from irregular word reading, forward digit span, DDT, and FPT.

### Clinical Implications

The cluster analysis does not provide any information on causal relationships, instead the purpose of the clusters is to determine common links and associations within groups of participants presenting with similar difficulties (i.e., listening complaints in the current study). Cluster 1 is the only group showing global difficulties across all domains. The remaining groups all have areas of strength as well as difficulties. The question arises – what makes Cluster 1 different. It is possible that the executive function is the missing link that may explain the poor performance overall of Cluster 1. In a recent paper Snowling et al. (2018) suggested that difficulties with executive control might explain the widely reported associations between language, reading and auditory processing difficulties. Partial correlations were not significant in this cluster and hence do not support a link such as this between these skills, however, this may be due to the relatively small sample in Cluster 1.

An alternative view is that all children (*N* = 90) within this cohort are similar and the differences in their profiles are due to strengths the children have developed, which could be compensatory or as a result of previous training or therapy. The children in the current study participated in the research when they were at least 7 years old. There were no reports of any injury or medical misadventure to account for the auditory processing concerns and, therefore, one can assume that all these children have a “developmental” APD (Moore et al., 2013). Could the current clusters be the consequence of individual compensatory mechanisms? At present, there are no empirical data to answer this question; however, future longitudinal research could consider this question regarding the effects of variations in intervention, neuroplasticity, and maturation on the profile of skills in children with auditory processing difficulties. According to the questionnaire completed by the parents, all children showed mild to extreme deficits on CHAPS, irrespective of their groups. A longitudinal study with intervention for younger children presenting with auditory processing difficulties (e.g., 5–6 year olds) might be the best way to determine validity of these clusters and to better understand the casual relation (if it exists) between cognitive skills including attention and memory, auditory processing, and language. Leppänen et al. (2010) found electrophysiological evidence for atypical processing of sound frequency in newborns who were later identified as having phonological, reading, and language difficulties.

Another noteworthy finding is the presence of poor FPT and DDT performance in the presence of good reading and phonological processing in Cluster 2. This is an important finding as it challenges the framework suggested by Tallal or Goswami that the auditory processing link to word reading is mediated by phonological processing (Ramus, 2003). It also challenges the proposal that executive control links language, auditory processing, and reading (Snowling et al., 2018). Overall, children in Cluster 2 showed good attention and memory skills. Cluster 2, therefore, appears to be a subgroup of children who have poor auditory processing skills not linked to reading, language, memory, or attention.

Children in Cluster 3 had poor FPT, attention, and memory scores in the presence of relatively good TONI, language, and reading skills. Based on structural equation modeling, Snowling et al. (2018) observed that executive function was predictive of frequency discrimination; therefore, it is possible that, as was the case for Cluster 1, this group could have poor executive function. The FPT test encompasses a range of skills, however, in addition to frequency discrimination, including pattern perception and verbal reporting skills, so this finding may be unrelated to the frequency discrimination aspect of the task.

Cluster 4 is somewhat similar to Cluster 3 as both groups exhibit poor performance on FPT and poor attention skills. However, Clusters 3 and 4 differ in their backward digit span scores, with Cluster 4 showing higher performance compared to Cluster 3. Poor attention is not an obvious explanation for poor auditory processing, as children in Cluster 2 had poor auditory processing despite the presence of good attention skills. While attention has been linked with performance on the AP tasks in general (Moore et al., 2010), sustained attention has not been found to contribute to the performance on the FPT (Gyldenkærne et al., 2014; Tomlin et al., 2015).

The participants in this study are likely to be representative of the children referred for clinical evaluation of auditory processing (since referrals to the research came from a range of professionals and parents). Consequently, the four clusters may be representative of children with suspected APD, but the identified clusters are unlikely to be the only ones that exist in the population of children with neurodevelopmental difficulties affecting learning and behavior. Despite this limitation, there are some potential benefits of identifying these subgroups of children presenting for auditory processing assessment. The distinct clusters identified in the current study highlight the heterogeneity of children with suspected APD, and this result encourages clinicians to ensure assessments span all the domains examined here, especially those that contributed most to the separation of the clusters, namely working memory (backward digit span), non-verbal IQ, non-word reading, and phonological processing. Assessment of these areas in children with a diagnosis of APD could assist clinicians to choose appropriate referral pathways and treatments.

Although all groups included children with APD and parent-rated listening difficulties based on the CHAPS questionnaire, some children might make better functional gains if their specific phonological processing, reading, and/or other difficulties are targeted. For instance, Cluster 1 might benefit from referral to a psychologist for cognitive assessment that includes measures of executive control and is likely to need a broad range of supports. Audiologists would best manage children in Cluster 2. Clusters 3 and 4 could benefit from auditory training delivered by an audiologist or speech pathologist and a psychologist would be able to conduct more comprehensive attention and memory assessment and management suggestions. Although this suggests a different pathway for each cluster, all children presented with listening difficulties, and hence all are likely to benefit from treatments such as personal remote microphone technology to improve the signal to noise ratio in difficult listening situations (Sharma et al., 2012).

Leppänen et al. (2010) identified auditory insensitivity 3–5 days after birth in about half of the infants they tested with familial risk for dyslexia, using a mismatch negativity paradigm (event-related potential response to infrequently presented 1100 Hz deviant sinusoidal stimulus among frequently presented 1000 Hz sinusoidal stimulus). They found that about half of the participants in this longitudinal study had impaired differentiation of basic pitch changes at birth and these children were later diagnosed with dyslexia; the other half of the children with normal mismatch negativity responses in infancy did not have problems in reading acquisition when tested 8 years later. This paper highlights the possibility of earlier identification of auditory difficulties using electrophysiological approaches. This would allow the possibility of early interventions targeted at enhancing auditory processing that might prevent later literacy difficulties. This could change the profiles of children with APD in the future.

### Limitations and Future Directions

Cluster analysis is an unbiased way to determine subgroups; there are some limitations, however. For instance, the participating 90 children created the current four clusters, and validation using a different, larger sample would be useful to confirm the characteristics of the clusters. With a larger sample, the details of the clustering might change (as in, some children could be assigned to a different cluster if the data looked a bit different, or different tests were included), but the overall differences between clusters identified in the current study are sufficiently pronounced that the interpretation of the subgroups identified here may not change. With a larger sample, the stability of the clusters could be determined by comparing the clustering of the original data set with the clustering obtained on subsamples or with a completely new data set (Levine and Domany, 2001).

The clusters did not differ in the balance of boys to girls (Chi-square = 1.31, *p* = 0.73), although there were more males than females overall. There was a trend for two clusters (Clusters 1 and 4) to be slightly younger [*F*(1, 3) = 3.02, *p* = 0.034] than the other two, however. Higher numbers with equal gender proportions to account for slight age and gender variations may assist in generalization of the clusters.

In the current cluster analysis only two auditory processing measures with established age norms were included (FPT and DDT). Inclusion of other auditory skills, such as spatial listening (LISN-S) (Cameron and Dillon, 2007) or temporal or frequency discrimination (Moore et al., 2010) might yield different results if these auditory skills are more strongly linked than FPT and DDT to cognition and other skill areas. In future research, it would be useful to include a wider range of norm-referenced auditory processing measures that capture the range of auditory skills typically included in the clinical auditory processing test battery. Due to the complexity of reading disorders (Horbach et al., 2019), a more detailed assessment of reading abilities and potential underlying deficits such as temporal or phonological processing might also affect cluster membership.

It is possible that children with neurodevelopmental disorders will show evidence of different difficulties at different ages, even if deficits were solely in the auditory domain at an early age. More longitudinal research is needed to establish the stability of clusters over time as it is possible that training of specific cognitive and/or auditory skills would give rise to different results over time.

## Data Availability Statement

The datasets analyzed in this manuscript are not publicly available. Requests to access the datasets should be directed to mridula.sharma@mq.edu.au.

## Ethics Statement

The studies involving the human participants were reviewed and approved by the University of Auckland Human Research Participants’ Ethics Committee. Written informed consent to participate in this study was provided by the participants’ legal guardian/next of kin and verbal assent from all participating children was also gathered.

## Author Contributions

MS: the concept of the project, data analysis, and writing of the manuscript. SP: the concept of the project and editorial of the manuscript. PH: advising on the data analysis and editorial of the manuscript.

## Conflict of Interest

The authors declare that the research was conducted in the absence of any commercial or financial relationships that could be construed as a potential conflict of interest.

## Abbreviations

APD: auditory processing disorder
TONI: test of nonverbal intelligence
PA: phonological awareness
FPT: Frequency Pattern Test
DDT: Dichotic Digits Test.
