# Supplementary material for: Cluster Analyses Reveals Subgroups of Children With Suspected Auditory Processing Disorders
Source: Front Psychol. 2019 Nov 15;10:2481. doi: 10.3389/fpsyg.2019.02481 (PMC6872645; doi:10.3389/fpsyg.2019.02481)
Supplement: Supplementary file 1 [file Table_1.docx]

**Supplementary table 1**. Discriminant analysis showing standardised coefficients for the three factors that assisted in determining the membership of the cases within the 4 clusters.

| Variables | Function 1 | Function 2 | Function 3 |
| --- | --- | --- | --- |
| Language |  | 0.31 | -0.30 |
| Phonological processing | 0.73 |  |  |
| Irregular word reading | -0.28 | 0.23 |  |
| Nonword reading | -0.25 | 0.31 | -0.53 |
| TONI |  | -0.85 |  |
| Auditory attention |  |  |  |
| Forward digit span |  |  | 0.36 |
| Backward digit span | -0.60 |  | 0.49 |
| DDT |  | 0.22 |  |
| FPT |  | 0.24 | 0.35 |

<0.20 standard coefficients were suppressed
